# Supplementary material for: Differences in visually induced MEG oscillations reflect differences in deep cortical layer activity
Source: Commun Biol. 2020 Nov 25;3:707. doi: 10.1038/s42003-020-01438-7 (PMC7688644; doi:10.1038/s42003-020-01438-7)
Supplement: Supplementary file 1 — Supplementary Information [file 42003_2020_1438_MOESM1_ESM.docx]

**Supplementary Material**

*Supplementary Methods*

*Mathematical proof that compartmental and neural mass models can predict similar brain data*

Below we include a mathematical justification of our approach. We show that after fitting the neural mass model to simulated data from the compartmental model we obtain a neural mass model which makes similar predictions about laminar dynamics as the compartmental model. This is because the parameters of the neural mass model are fine tuned to produce similar data as the compartmental model. “Similar” has a precise, mathematically rigorous, meaning. We will see below that it means that a Bayesian observer can not distinguish between the data predicted separately by each model. Thus, the models from the point of view of a Bayesian observer predict the same data. Alternatively, this means that if one fits both models to the same data using Bayesian inference then the model fits have the same error or accuracy.

During this inference process, the parameters of the two models were obtained by applying decision rules that are equivalent, that is, they belong to the same admissible class. This mathematical equivalence follows from reformulating the inference problem (parameter estimation), as a decision problem. Reformulating parameter inference in the context of statistical decision theory is a well-established approach, see for instance [36], [37]. Mathematically, this means that parameter inference is *not* based on the optimization of a likelihood function but that of a *loss* function instead.

To sum up, our analysis below is based on reformulating parameter inference as the following statistical decision problem: find the true states of nature (biophysical parameters $\vartheta$ of a model *M*) that generate neural activity in different cortical depths after observing non-invasive (M/EEG) brain data.

To address this problem we first need to introduce notions from statistical decision theory. Of course, the following is not meant to be an exhaustive tutorial. For this, we refer the interested reader to [7]. Consider a sample space $O$, that is a Borel subset of a Euclidean space, and an observation or sample of brain data . Assume that the underlying cortical circuitry can be described by the model *M* with parameters (also called latent or true states) , *m<n,* where $\Theta$is called the space of all possible parameters. Since $\vartheta$ parameterize *M* which is a biophysical model, the space $\Theta$ includes all biophysically realistic model parameters*.*  In the context of statistical decision theory, a decision rule $\delta$ is the rule (or method) that ­the statistician uses to infer the parameters $\vartheta$ based on brain data $o\in O.$ The estimates of the parameters $\vartheta$ are denoted by . The notation $a=a(o)$ is often used to denote that they depend on the data $o.$Statistical decision theory says that there are many decision rules from which these estimates can be obtained. These give rise to the space $A$, the space of possible estimates, called also the decision space. Each different rule gives a different estimate of $\vartheta$. To sum up, the decision rule $\delta$ allows the statistician to solve the inference problem of estimating the parameters $\vartheta$ using observations $o\in O$*.* The rule allows the statistician to decide what the true value of $\vartheta$ might be. In mathematical terms, the decision rule is a mapping from the sample space $O$ into the decision space$A$, that is, $\delta$ is such that $\delta:O\to^{\delta} A$ .

Consider now that $\vartheta=(\vartheta_{1},\vartheta_{2},...,\vartheta_{q})$ are the parameters of a biophysical model, e.g. a neural mass model. The neural mass model is a *generative* model of brain data. Mathematically, a generative model is a mapping from biophysical parameters $\vartheta$ to observed data $o\in O$. It is defined by the conditional probability of observing a brain data $o$ given a parameters (latent states) $\vartheta$, that is $p(o\left|\vartheta\right.).$ In the context of statistical decision theory, biophysical parameter inference can be thought of as a decision problem where using the decision rule $\delta$, we obtain an estimate of model parameters. This is collectively denoted by $a=(a_{1},a_{2},...,a_{q})$. In brief, the true and estimated model parameters are denoted by $\vartheta_{k}$and $a_{k}$ respectively, and $k\in\{1,...,q\}$runs over all model parameters $\vartheta_{k}$. Also, the probability distribution $p(\vartheta)$ quantifies the belief of the statistician in the likelihood that the model parameters are equal to $\vartheta$, *before* observing $o\in O$.

The error the statistician will make while inferring the parameters using the decision the rule $\delta$ can be quantified in terms of two functions: the loss and expected loss (also known as risk) functions. Statistical decision theory says that for any rule $\delta$, we can define the *loss function* as a real valued function that quantifies the discrepancy between the true and estimated values of each model parameter. The *expected* value of the loss function under$p(\vartheta,a)$ , (where $p(\vartheta,a)$ is the prior that quantifies the belief of the statistician that the model parameters are equal to $\vartheta$, and the parameter estimates are equal to $a$ *before* making any observations $o\in O$), is called *expected loss* $C(\vartheta,a)$

$C(\vartheta,a)=\iint L(\vartheta,a)p(\vartheta,a)d\vartheta da$ (1)

We will here consider that the statistician uses a particular kind of inference, that is, Bayesian inference. This is because DCM that we used for parameter estimation uses Bayesian inference. To sum up, after defining the loss and expected loss functions above, we now turn to Bayesian inference. This means that the decision rule is also of a particular kind, that is, Bayesian. Using a Bayesian rule (and inference) we obtain Bayesian estimates denoted by tilde, $\tilde{a}\in A$. These are distributed according to $p(\tilde{a}\left|\vartheta\right.)$. The uncertainty in our estimates is included in the probability distribution $p(\tilde{a}\left|\vartheta\right.)$. This quantifies the belief that the model parameters are equal to $\tilde{a}$ *after* observing $o\in O$. The expected value of the loss function $L(\vartheta,\tilde{a})$ *after* obtaining Bayesian estimates $\tilde{a}=(\tilde{a}_{1},\tilde{a}_{2},...,\tilde{a}_{q}),$ is called *Bayes loss* and is given by

$R(\vartheta,\tilde{a})=\int L(\vartheta,\tilde{a})p(\tilde{a}\left|\vartheta\right.)d\tilde{a}$ (2)

In other words, the Bayes loss $R(\vartheta,\tilde{a})$ quantifies the expectation of the statistician about the error they will make when using Bayesian inference.

There are many ways to implement Bayesian inference, each corresponding to a different Bayesian decision rule $\tilde{\delta}$. For example, one could obtained maximum a posteriori (MAP) or maximum likelihood (ML) estimates. The *common property of all Bayesian rules* (the mathematical requirement that defines a decision rule as Bayesian) is that it minimizes the expected loss $C(\vartheta,a)$ [7]. This also means that any decision rule that is not Bayesian fails to minimize the expected loss[[1]](#footnote-1). In mathematical terms, this is written as follows: a Bayesian decision rule $\tilde{\delta}$ is defined by the requirement that the parameter estimates $\tilde{a}=(\tilde{a}_{1},\tilde{a}_{2},...,\tilde{a}_{q})$ are such that $\text{ }C(\vartheta,\tilde{a})\text{ }\equiv\mathop{\text{inf}}\limits_{\delta:o\in O\to a\in A} \text{ }C(\vartheta,a)$. For Bayesian decision rules, the expected loss $C(\vartheta,\tilde{a})$ and Bayes loss $R(\vartheta,\tilde{a})$ are related as follows

$C(\vartheta,\tilde{a})=\int R(\vartheta,\tilde{a})p(\vartheta)d\vartheta$ (3)

In other words, for Bayesian decision rules, the expected loss is just the expected value of the Bayes loss under the prior $p(\vartheta)$ . Also, a famous theorem in statistical decision theory known as the *complete class theorem* [7] says that all Bayes rules are *admissible* that is, they all have the *same* expected loss. For example, assume two such rules $\tilde{\delta}_{1}$and $\tilde{\delta}_{2}$with corresponding estimates (of the same parameters $\vartheta$), $\tilde{a}=(\tilde{a}_{1},\tilde{a}_{2},...,\tilde{a}_{q})$ and $\tilde{b}=(\tilde{b}_{1},\tilde{b}_{2},...,\tilde{b}_{q})$. Then, $C(\vartheta,\tilde{a})=C(\vartheta,\tilde{b})$.

We show below that this property of Bayesian decision rules has interesting implications for Bayesian estimates of parameters from *different* biophysical *models* that describe the *same* *brain* *network*. Consider two models, model *M1* and model *M2*, describing the brain network activated by the stimulus in our task[[2]](#footnote-2). This is shown in Figure 1. Let us denote the model parameters of *M1* and *M2* by $\vartheta=(\vartheta_{1},\vartheta_{2},...,\vartheta_{q})$and respectively. After measuring brain data $o\in O$, Bayesian parameter estimates of *M1* and *M2* are denoted by $\tilde{a}_{_{\vartheta}}=(\tilde{a}_{\vartheta_{1}},\tilde{a}_{\vartheta_{2}},...,\tilde{a}_{\vartheta_{q}})$ and . Here, *M1* is a neural mass model and *M2* is a compartmental model describing the same neuronal circuitry depicted in Figure 1. Both models describe the same brain area, visual cortex in our work, but with *different spatial resolution*. *M2* includes a description of microstructure like dendrites, axons, synapses etc. that span different cortical depths, while *M1* describes neuronal populations, their dynamics, connectivity etc at a coarser spatial scale. Because the compartmental model describes microstructure the number of its parameters is much larger, that is, *r>q*. From a practical point of view, this means that while parameters of *M1* can be estimated efficiently using Bayesian inference, parameters of *M2* cannot (e.g. spiking model parameters from individual neurons cannot be inferred from LFPs).

Below, we construct a variant of model *M2*  with the same number of parameters as *M1, q*. Virtual LFP electrode responses $y(t,\theta)$ predicted by the neural mass model *M1* are generated by contributions from excitatory and inhibitory populations that occupy one or more cortical layers (Figure 1). The relationship between $y(t,\theta)$ and depolarization $v_{m}(t)$at each layer *m* is described by the evolution equations of the model and the forward mapping linking neural responses to LFPs [8]

(4)

Here, is a lead field describing the spatial sensitivity of a virtual sensor to activity in layer *m*, $\theta=(\psi,\vartheta)$are lead field and neural mass model parameters respectively and $\sigma$ is a sigmoid operator transforming it into firing rate. $\kappa_{m}$ is the matrix of rate constants associated with postsynaptic processing and *U* stands for the inputs to local cortical circuit, see [8] for more details.

For finding the parameters of the neural mass model *M1* used here, we first source reconstructed MEG data to obtain $y(t,\theta)$. We then fitted *M1* to power spectra $g_{Y}(\omega)_{i}$that can be straightforwardly obtained from $y(t,\theta)$. Similarly to the neural mass model *M1,* the compartmental model *M2* describes activity from the cortical circuit depicted in Figure 1. However, it describes this activity in more detail as generated by an ensemble of smaller structures called mini-columns. Below we considered ten mini-columns. These comprise the macro-column shown in Figure 1. In this setting, activity predicted from the compartmental model *M2* (see [5]) is a simple superposition of minicolumn activities $Y_{a}(t,\theta)$

(5)

where the index $q'\subseteq q$ runs over a subset of *compartments* $q'$ that comprise each mini-column and $a=1,...,10$ runs over the mini-columns. Each mini-column comprises the compartments of superficial PN, deep PN and superficial and deep interneurons, and $Q(J_{k},L_{k},c_{aa'},\xi)$ stands for the exogenous input – that depends on activity in proximate compartments indexed by$k\in\{q\},k\neq q'$*.* The argument in the factor *Q* in Equation (5) above simply means that this input depends upon the current density in the adjacent mini-columns, their lead fields, anatomical parameters $\xi$ and the strength of connections $c_{aa'}$.

We assumed that $c_{aa'}$are the same between any mini-column pair and that all mini-columns have the same anatomy. Thus, the number of parameters in model *M2* was reduced by a factor equal to the number of minicolumns comprising one macrocolumn. Because *M2* describes the same circuit as *M1* and all minicolumns have the same parameters, the resulting model (which we call, the *symmetric compartmental model* and denote by *M2****’***) has the same number of connection parameters as the neural mass model *M1*. We denote the *M2’* parameters by and the corresponding Bayesian estimates by . Despite the reduction in the number of parameters, predictions of laminar dynamics from models *M2* and *M2’* were very similar, see [8]. The macrocolumn generates steady-state oscillations, and all microcolumns receive the same input. Synchronized minicolumn activity lies on an invariant subspace in the full phase space of the network; see [38], [39] for a discussion of invariant subspaces. At the same time, this neglects horizontal interactions within the same cortical layer at the expense of describing laminar interactions with a neural mass model that can then be fitted to non-invasive MEG data. This is achieved by combining model *M2****’*** with *M1.* We will come back to it below. To sum up, we assumed that all mini-columns in the compartmental model *M2* have the same anatomy and the connection weights $c_{aa'}$are the same between any mini-column pair. How are then the expected and Bayes losses (cf. Equations 1 and 2) associated with *M1* and *M2’* parameters connected? Because *M1* and the symmetric model *M2’* have the same number of parameters and describe the same neural circuitry and brain data, the Bayesian estimates of their parameters satisfy

(6)

This follows from the complete class theorem [7]. The Bayesian parameter estimates of the two models have the same expected loss. Also, the corresponding Bayes loss satisfies (after combining Equations (3) and (6) )

(7)

Besides predicting the same brain dynamics, we are also interested in models that can describe the brain network equally well. This implies that the prior belief of the statistician about the true model parameters is the same for both *M1* and *M2’*, . Then Equations (7) and (2) yield

(8)

Interestingly, it turns out that models *M1* and *M2’* have the same *maximum a posteriori* *(MAP)* estimates. This follows from the above Equation (8). Assuming point density probability distributions $p(\tilde{a}_{\vartheta}\left|\vartheta\right.)$ and , we obtain an equality between the *MAP* estimates $\mu(\tilde{a}_{\vartheta}\left|\vartheta\right.)$ and of parameters of the two models *M1* and *M2’*

(9)

This is our first mathematical result:

*Result 1.* *The MAP estimates* $\mu(\tilde{a}_{\vartheta}\left|\vartheta\right.)$ and  *of parameters $\vartheta$and* *of M1 and M2’ obtained using the same data* $o\in O$ *are equal.*

In the following, we focus on estimating *M1* (neural mass) model parameters by fitting it to MEG source reconstructed data, using Bayesian inference and the *Result 1* above. We here use DCM to implement Bayesian inference. DCM is based on an EM algorithm to obtain the posterior distribution of *M1* model parameters $p(\tilde{a}_{\vartheta}\left|\vartheta\right.)$ by optimizing the free energy, $F(o,\vartheta),$ see e.g. [40] for details:

$\begin{matrix} p(\tilde{a}_{\vartheta}\left|\vartheta\right.)=\arg\min_{\tilde{a}_{\vartheta}} F(o,\vartheta) \\ F(o,\vartheta)=-KL(q(\tilde{a}_{\vartheta}\vartheta)\left|p(\right.\vartheta))+\int\log p(o\left|\tilde{a}_{\vartheta},\vartheta\right.)q(\tilde{a}_{\vartheta}\left|\vartheta\right.)d\vartheta\end{matrix}$ (10)

where $p(o\left|\tilde{a}_{\vartheta},\vartheta\right.)$ is the data log-likelihood and $q(\tilde{a}_{\vartheta}\left|\vartheta\right.)$is the approximate posterior. The above is often called a maximum likelihood (ML) optimization problem. Here, we rewrite it as

$\begin{matrix} p(\tilde{a}_{\vartheta}\left|\vartheta\right.)=\arg\min_{\tilde{a}_{\vartheta}} \left[f(\vartheta)+f(o)\right] \\ f(\vartheta)=-KL(q(\tilde{a}_{\vartheta}\left|\vartheta\right.)\left|p(\right.\vartheta)) \\ f(o)=\int\log p(o\left|\tilde{a}_{\vartheta},\vartheta\right.)q(\tilde{a}_{\vartheta}\left|\vartheta\right.)d\vartheta\end{matrix}$ (11)

where we have simply substituted $F(o,\vartheta)$ by

$F(o,\vartheta)=f(\vartheta)+f(o)$ (12)

to emphasise that while optimizing $F(o,\vartheta)$we are constraining the posterior distribution by exploiting information in both our prior beliefs about the model parameters $p(\vartheta)$ and also the observations $o\in O$. Thus the posterior should not move too far from our prior guess and also will maximize the data log-likelihood. This reformulation also allows us to establish a connection between Bayesian inference based on free energy optimization and statistical decision theory: free energy can be thought of as a cumulative loss function of the form of Equation (12). Statistical decision theory regards Bayesian inference as a decision problem that optimizes this loss function. The statistician collects observations $o\in O$ to infer parameters. Assume that we sample the data space successively $O$, obtaining brain data $o_{1}$first, then $o_{2}$,$o_{3}$ etc. with $o_{1},o_{2},o_{3},...\in O.$ Bayesian belief updating suggests that that we can assume that the loss function (12) can optimized in the same successive, optimal and rational manner after observing each data sample $o_{i}\in O$, *i=1,2,…*, that is $F(o,\vartheta)=\sum_{i} \left(f_{i}(\vartheta)+f_{i}(o)\right)$ . Let us then focus on using the *first sample* $o_{1}$to obtain *M1* model parameters $\vartheta$. To use *Result 1* above and connect predictions from *M1* and *M2’,* $o_{1}$is chosen to be *simulated data from M2’* (the symmetric compartmental model).

Consider also the ML optimization problem given by Equation (11) for $o_{1}$:

$\begin{matrix} p(\tilde{a}_{\vartheta}^{1}\left|\vartheta\right.)=\arg\min_{\tilde{a}_{\vartheta}} \left[f_{1}(\vartheta)+f_{1}(o^{1})\right] \\ f_{1}(\vartheta)=-KL(q(\tilde{a}_{\vartheta}^{1}\left|\vartheta\right.)\left|p(\right.\vartheta)) \\ f_{1}(o^{1})=\int\log p(o^{1}\left|\tilde{a}_{\vartheta}^{1},\vartheta\right.)q(\tilde{a}_{\vartheta}^{1}\left|\vartheta\right.)d\vartheta\end{matrix}$ (13)

where we use an upper index, *1*, in $\tilde{a}^{1}_{\vartheta}$to denote the parameter estimates obtained after observing the first data sample $o_{1}$.

Recall now that the MAP estimates for the *M1* and *M2’* are equal , that is, and we assumed that *M1* and *M2’* describe the cortical microcircuit equally well, i.e. . Assuming also uniform priors, the ML optimization problem (15) is the same as the MAP estimation problem we considered earlier. Thus we can replace $\vartheta$ by in Equation (13) and obtain

(14)

Noting that in this case $f_{1}(o^{1})=0$ ( because we used simulated data) the above Equation (16) yields the ML optimization problem for *M2’*

(14’)

In brief, we started with the ML optimization problem for model *M1* and *showed that it is the same as* the ML optimization problem for model *M2’.* This is our second mathematical result.

*Result 2.* *The ML problems for models M1 and M2 given by Equations (13) and (14’) are the same.* This means that fitting the neural mass (*M1*) model to simulated data $o_{1}$from the compartmental model (*M2’*), we perform the same parameter inference as if we were fitting the compartmental model itself.

Based on this result we argue that the neural mass model whose parameters have been fitted to simulated data from the compartmental model contains the same information as the compartmental model. Having established the equivalence of inference problems for the first data sample $o_{1}\in O$ , we consider another sample $o_{2}\in O$. This is the MEG source reconstructed data. We fit model *M1* to $o_{2}$. Similarly to Equation (13), this inference problem for sample $o_{2}$ can be written as

$\begin{matrix} p(\tilde{a}_{\vartheta}^{2}\left|\vartheta\right.)=\arg\min_{\tilde{a}_{\vartheta}} \left[f_{2}(\vartheta)+f_{2}(o^{2})\right] \\ f_{2}(\vartheta)=-KL(q(\tilde{a}_{\vartheta}^{2}\left|\vartheta\right.)\left|p(\right.\vartheta)) \\ f_{2}(o^{2})=\int\log p(o^{2}\left|\tilde{a}_{\vartheta}^{2},\vartheta\right.)q(\tilde{a}_{\vartheta}^{2}\left|\vartheta\right.)d\vartheta\end{matrix}$ (15)

where using *Result 1,* the expectation of $p(\vartheta)$, $<p(\vartheta)>=$. In other words, it is equal to the MAP estimate obtained from the previous step (fitting the simulated data $o_{1}$). To sum up, instead of fitting the neural mass model *M1* to the MEG data $o_{2}\in O$ (as done in traditional DCM), we considered an extended data space comprising simulated and empirical data $o_{1},o_{2}\in O$ and successively fitted this to $o_{1}$ and $o_{2}$. The first fit ensured that model *M1* has the same construct validity (makes the same predictions) as the compartmental model *M2’*. The second fit adjusted laminar predictions from *M1* to explain MEG data.

**Supplementary Figures**

**
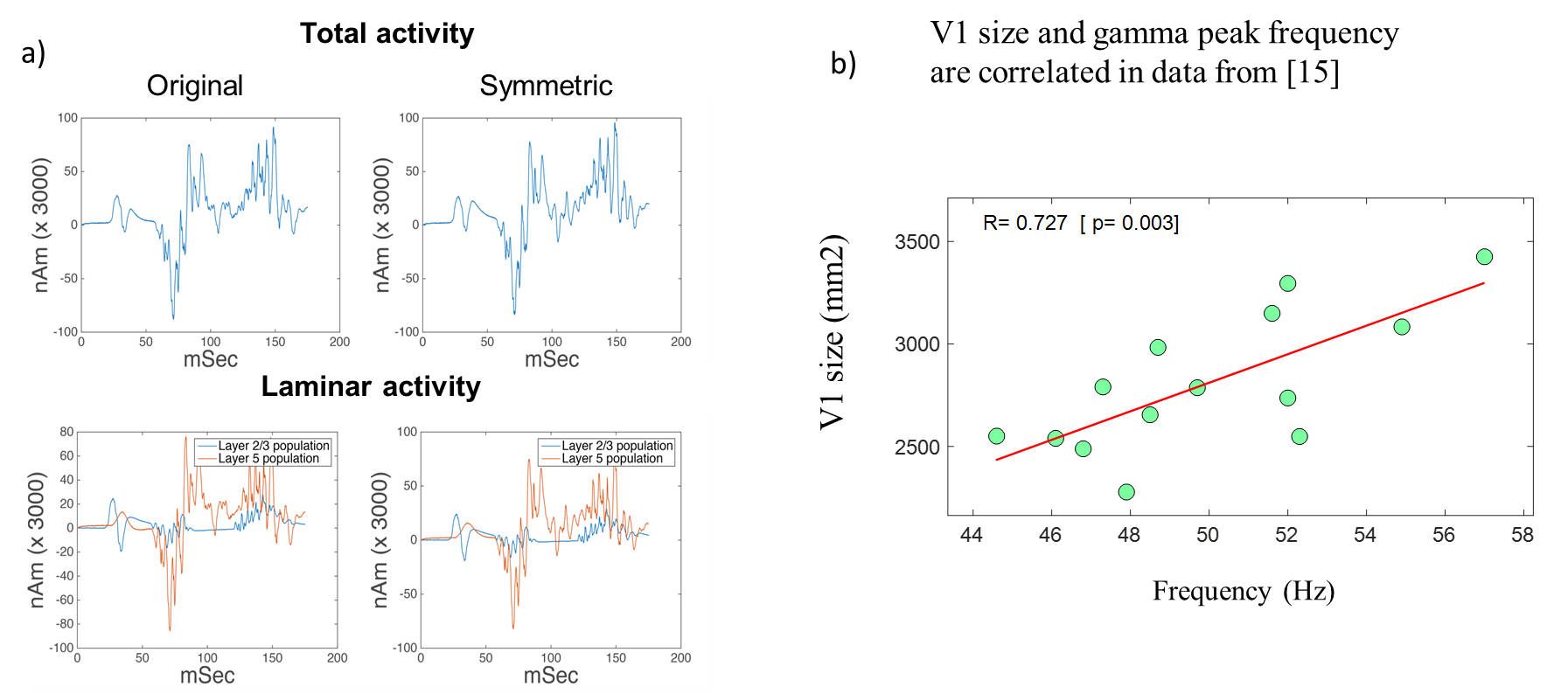
**

**Fig. S1. a)** (Top) Simulated evoked responses of (left) the compartmental models; (right) its symmetric variant. (Bottom) Contributions to the net dipole per layer for the same models; see [8] for more details. **b)** Strong positive correlation between the size of primary visual cortex in a certain subject and the corresponding gamma peak frequency (*R=0.727, p=0.003*) reported in [15]. Data from two subjects among the original pool were discarded due to poor quality of the corresponding V1 size measurement. Least squares fitted line shown in red.


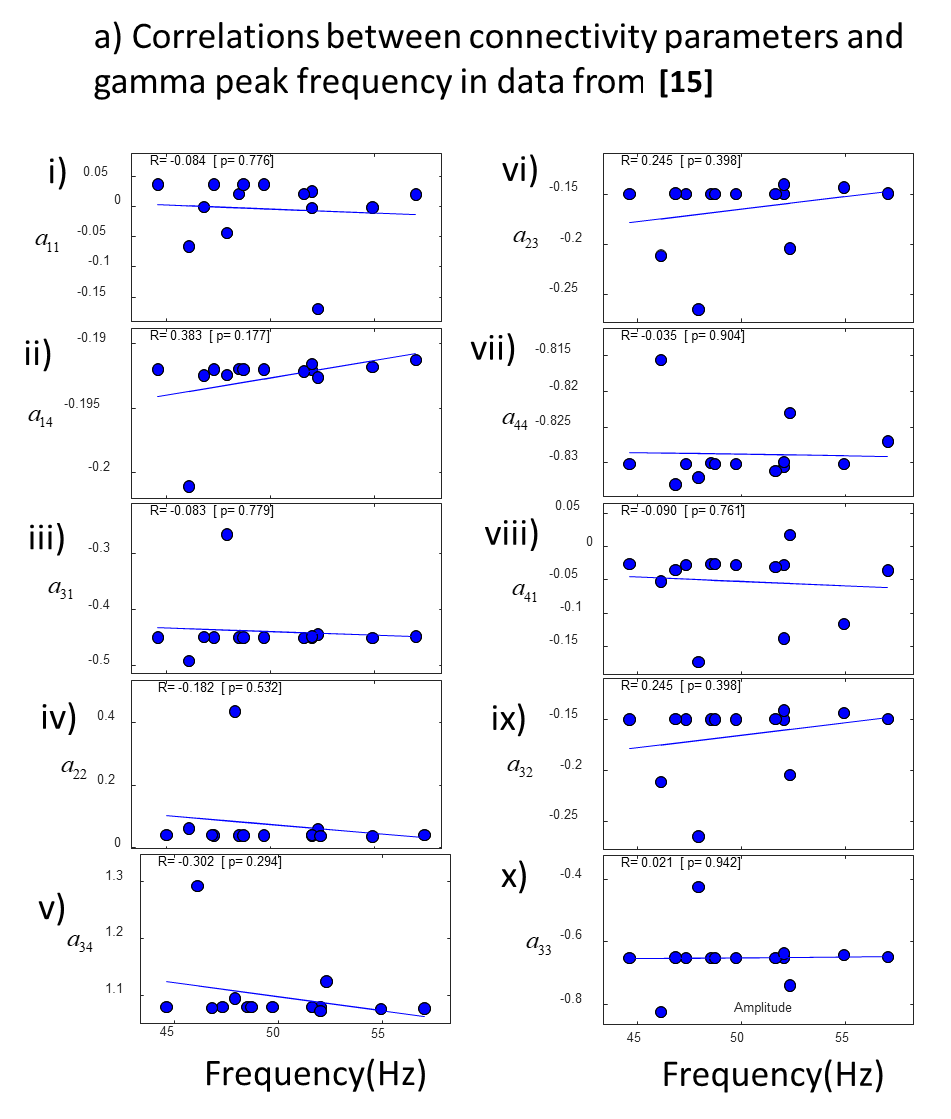


**Fig. S2. a)** Non significant correlations between the connectivity parameters appearing in Figure 1A and gamma peak frequency. Parameters we obtained after fitting the neural mass model to data from [15]. Least squares fitted line shown in blue.

**
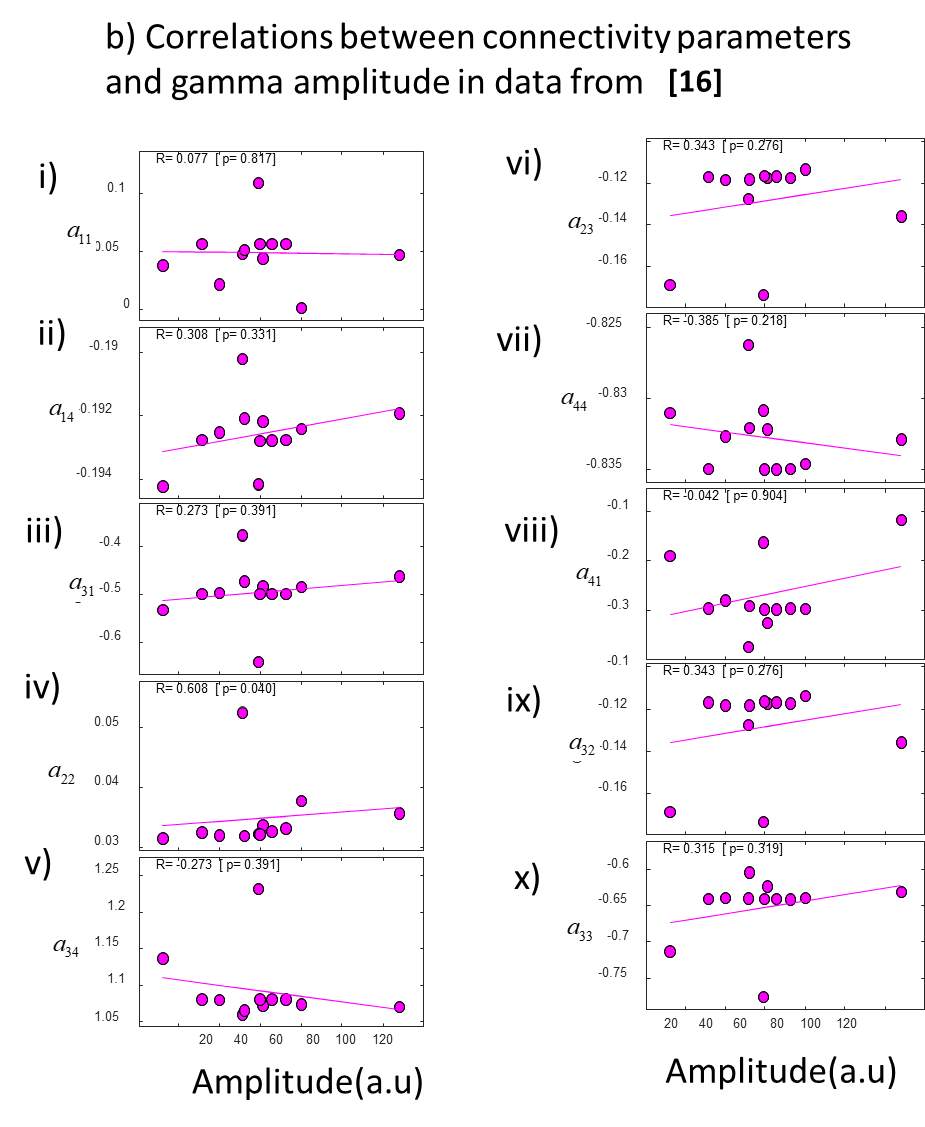
**

**Fig. S2. b)** Correlations between connectivity parameters and gamma amplitude in data from [16]. Changes in recurrent connections in deep inhibitory interneurons, $a_{22}$,correlated with changes in gamma amplitude. Least squares fitted line shown in magenta.

**
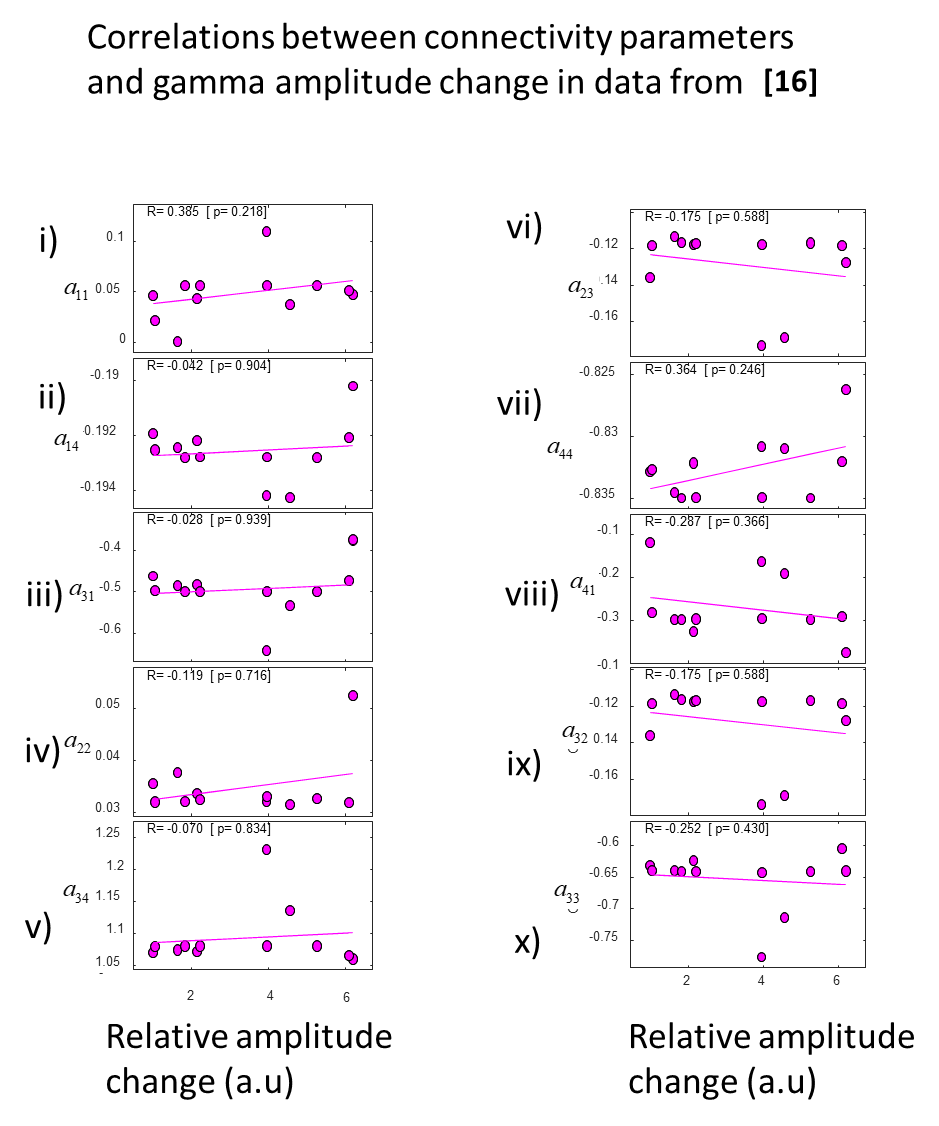
**

**Fig. S3.** Non significant correlations between the connectivity parameters appearing in Figure 1A and relative gamma amplitude change. Parameters we obtained after fitting the neural mass model to data from [16]. Least squares fitted line shown in magenta.

1. Note that in the abstract formulation of statistical decision theory, explicit use of Bayes theorem is not required to define Bayes rules (the definition covers approximate Bayesian inference too). For more details, see (Berger, 2013). [↑](#footnote-ref-1)
2. These models are *a priori equally plausible* models of the *same* observed brain dynamics $o\in O$. [↑](#footnote-ref-2)
