# Supplementary material for: Differences in visually induced MEG oscillations reflect differences in deep cortical layer activity
Source: Commun Biol. 2020 Nov 25;3:707. doi: 10.1038/s42003-020-01438-7 (PMC7688644; doi:10.1038/s42003-020-01438-7)
Supplement: Supplementary file 3 — Reporting Summary [file 42003_2020_1438_MOESM3_ESM.pdf]

nature research

Corresponding author(s): Dimitris Pinotsis

Last updated by author(s): May 25, 2020

Reporting Summary

Nature Research wishes to improve the reproducibility of the work that we publish. This form provides structure for consistency and transparency in reporting. For further information on Nature Research policies, see our Editorial Policies and the Editorial Policy Checklist.

Statistics

For all statistical analyses, confirm that the following items are present in the figure legend, table legend, main text, or Methods section.

n/a	Confirmed	
☐	☒	The exact sample size (n) for each experimental group/condition, given as a discrete number and unit of measurement
☐	☒	A statement on whether measurements were taken from distinct samples or whether the same sample was measured repeatedly
☒	☐	The statistical test(s) used AND whether they are one- or two-sided<br>*Only common tests should be described solely by name; describe more complex techniques in the Methods section.*
☒	☐	A description of all covariates tested
☒	☐	A description of any assumptions or corrections, such as tests of normality and adjustment for multiple comparisons
☒	☐	A full description of the statistical parameters including central tendency (e.g. means) or other basic estimates (e.g. regression coefficient) AND variation (e.g. standard deviation) or associated estimates of uncertainty (e.g. confidence intervals)
☒	☐	For null hypothesis testing, the test statistic (e.g. F, t, r) with confidence intervals, effect sizes, degrees of freedom and P value noted<br>*Give P values as exact values whenever suitable.*
☐	☒	For Bayesian analysis, information on the choice of priors and Markov chain Monte Carlo settings
☒	☐	For hierarchical and complex designs, identification of the appropriate level for tests and full reporting of outcomes
☐	☒	Estimates of effect sizes (e.g. Cohen's d, Pearson's r), indicating how they were calculated

Our web collection on statistics for biologists contains articles on many of the points above.

Software and code

Policy information about availability of computer code

Data collection	We here re-analysed data whose collection and first analysis is described in the earlier publications of (Schwarzkopf et al., 2012) and (Perry et al., 2013).
Data analysis	This uses the DCM toolbox of SPM12 https://www.fil.ion.ucl.ac.uk/spm/software/spm12/, and custom based code that can be found at https://github.com/pinotsislab/MicroMacro/ This code is based on SPM and the NEURON programming language https://www.neuron.yale.edu/neuron

For manuscripts utilizing custom algorithms or software that are central to the research but not yet described in published literature, software must be made available to editors and reviewers. We strongly encourage code deposition in a community repository (e.g. GitHub). See the Nature Research guidelines for submitting code & software for further information.

Data

Policy information about availability of data

All manuscripts must include a data availability statement. This statement should provide the following information, where applicable:

- Accession codes, unique identifiers, or web links for publicly available datasets
- A list of figures that have associated raw data
- A description of any restrictions on data availability

The data that support the findings of this study are available from the corresponding author upon reasonable request

Field-specific reporting

Please select the one below that is the best fit for your research. If you are not sure, read the appropriate sections before making your selection.

☐ Life sciences ☒ Behavioural & social sciences ☐ Ecological, evolutionary & environmental sciences

For a reference copy of the document with all sections, see nature.com/documents/nr-reporting-summary-flat.pdf

Behavioural & social sciences study design

All studies must disclose on these points even when the disclosure is negative.

Study description	Quantitative
Research sample	We used two datasets to test whether our model yields similar results in datasets taken from similar but independent experiments. First, we used visually induced oscillations taken from [16], where whole-head MEG recordings were acquired from 12 healthy subjects. In the second dataset, we used MEG beamformed data from [15]. These included visually induced oscillations from the visual cortex of 16 subjects.
Sampling strategy	Convenience
Data collection	MEG recordings
Timing	This is a meta-analysis of data originally reported in (Schwarzkopf et al., 2012) and (Perry et al., 2013).
Data exclusions	please see above
Non-participation	please see above
Randomization	please see above

Reporting for specific materials, systems and methods

We require information from authors about some types of materials, experimental systems and methods used in many studies. Here, indicate whether each material, system or method listed is relevant to your study. If you are not sure if a list item applies to your research, read the appropriate section before selecting a response.

Materials & experimental systems

n/a	Involved in the study	
☒	☐	Antibodies
☒	☐	Eukaryotic cell lines
☒	☐	Palaeontology and archaeology
☒	☐	Animals and other organisms
☐	☒	Human research participants
☒	☐	Clinical data
☒	☐	Dual use research of concern

Methods

n/a	Involved in the study	
☒	☐	ChIP-seq
☒	☐	Flow cytometry
☒	☐	MRI-based neuroimaging

Human research participants

Policy information about studies involving human research participants

Population characteristics	This is a meta-analysis of data originally reported in (Schwarzkopf et al., 2012) and (Perry et al., 2013).
Recruitment	please see above
Ethics oversight	please see above

Note that full information on the approval of the study protocol must also be provided in the manuscript.
